# Vitamin Status as a Determinant of Serum Homocysteine Concentration in Type 2 Diabetic Retinopathy

**DOI:** 10.1155/2014/807209

**Published:** 2014-06-10

**Authors:** Pandelis Fotiou, Athanasios Raptis, George Apergis, George Dimitriadis, Ioannis Vergados, Panagiotis Theodossiadis

**Affiliations:** ^1^2nd Department of Ophthalmology, “Attikon” University Hospital, Athens University Medical School, 1 Rimini Street, 12462 Athens, Greece; ^2^2nd Department of Internal Medicine-Propaedeutic, Research Institute and Diabetes Center, “Attikon” University Hospital, Athens University Medical School, 1 Rimini Street, 12462 Athens, Greece; ^3^Department of Molecular Diagnosis, “Hippokration” General Hospital, 114 Vasilissis Sofias Avenue, 11527 Athens, Greece; ^4^Athens Eye Hospital, 45 Vouliagmenis Avenue, 16675 Athens, Greece

## Abstract

We investigated the association of serum homocysteine levels and vitamin status with type 2 diabetic retinopathy. This study included 65 patients with and 75 patients without diabetic retinopathy. Patients with diabetic retinopathy had significantly higher serum homocysteine levels (*P* < 0.001), higher prevalence of hyperhomocysteinemia (*P* < 0.001), lower serum folic acid (*P* < 0.001), and vitamin B_12_ (*P* = 0.014) levels than those without diabetic retinopathy. Regression analysis revealed that homocysteine was an independent risk factor for diabetic retinopathy and there was a threshold in its serum level (13.7 **μ**mol/L), above which the risk of diabetic retinopathy greatly increases (OR = 1.66, *P* = 0.001). Folic acid was associated with decreased odds for diabetic retinopathy (OR = 0.73, *P* < 0.001). There was a threshold in serum vitamin B_12_ level (248.4 pg/mL), below which serum homocysteine concentration significantly increases with decreasing serum vitamin B_12_ (*P* = 0.003). Our findings suggest that hyperhomocysteinemia is an independent risk factor for the development and progression of diabetic retinopathy. Decreased serum levels of folic acid and vitamin B_12_, through raising serum homocysteine concentrations, may also affect the diabetic retinopathy risk.

## 1. Introduction

Progressive disorders of the large and small vessels are common and serious complications of diabetes mellitus (DM). Studies over the last two decades have shown that hyperhomocysteinemia is associated with several macrovascular diseases, such as coronary artery disease, cerebrovascular disease, peripheral arterial disease, and deep-vein thrombosis [1–3].

Relatively few studies have investigated the association between hyperhomocysteinemia and diabetic retinopathy (DR) with yet inconsistent results. In addition, the vitamin status and its contribution to the development of DR have not been examined. In our study, we aimed to investigate whether there is an association between serum levels of homocysteine and those of folic acid and B-vitamins with DR in patients with type 2 diabetes mellitus (T2DM).

## 2. Materials and Methods

### 2.1. Subjects

The study group included 140 patients with T2DM, 69 men and 71 women, aged from 41 to 83 years (mean age 64.4 ± 9.9 years), all of Greek descent. All patients were recruited from the Ophthalmology Outpatient Clinics as well as the Diabetes Center of the “Attikon” University Hospital (Medical School of Athens University) in Athens, Greece. Diabetes was diagnosed according to the guidelines of the American Diabetes Association [4].

A detailed medical history was obtained from all study participants. The study included patients with known age of diagnosis of diabetes >40 years. We excluded patients with one or more of the following conditions:

1. patients with a history of vascular disease (myocardial infarct or angina, stroke, peripheral arterial disease, and deep-venous thrombosis), renal, hepatic, chronic gastroenterologic, thyroid or blood disease, dementia, and neoplasm, since all these conditions are known to affect homocysteine blood concentrations;
2. patients receiving vitamin supplementation or medications known to affect serum homocysteine concentrations, such as theophylline, statins, fibrates, levodopa, protons pump inhibitors, anticonvulsives, and contraceptives, and patients consuming good amounts of alcohol and heavy smokers;
3. patients with uncontrolled arterial hypertension, defined as a systolic blood pressure ≥ 140 mmHg, and/or a diastolic blood pressure ≥ 90 mmHg;
4. patients with uncertain diabetes duration.

The study was in accordance with the declaration of Helsinki and was approved by the Local Ethics Committee. Written informed consent was obtained from all subjects.

### 2.2. Methods

#### 2.2.1. Ophthalmologic Examination

All patients underwent a complete ophthalmologic examination. Retinopathy was assessed by dilated ophthalmoscopy, fundus photography, and fluorescein angiography, when was indicated. Retinopathy was defined as the presence of any of such characteristic lesions: microaneurysms, hemorrhages, cotton wool spots, intraretinal microvascular abnormalities, hard exudates, venous beading, new vessels, laser scars, or a history of vitrectomy. Grading of the retinal findings was assessed according to the system used by the Early Treatment Diabetic Retinopathy Study Research Group [5]. Patients with diabetes were classified into two groups according to the findings of the “worst eye”: 65 with DR (46.4%) and 75 without DR (53.6%). Of the 65 patients with DR, 36 (55.4%) had nonproliferative diabetic retinopathy (NPDR) and 29 (44.6%) had proliferative diabetic retinopathy (PDR).

#### 2.2.2. Biochemical Analyses

Overnight fasting blood samples were taken from all subjects and were centrifuged within 1 hour following collection. The samples for homocysteine estimation were immediately chilled on ice and then were centrifuged and the serum was stored at −20°C. Serum glucose concentration was measured with a colorimetric assay on an automated analyzer MODULAR P800 (Roche Diagnostics GmbH, Mannheim, Germany) and glycosylated hemoglobin (HbA_1C_) with an immunoturbidimetric assay on an analyzer INTEGRA 400plus (Roche Diagnostics GmbH, Mannheim, Germany). Serum creatinine concentration was measured with the colorimetric assay (Jaffé) on the automated analyzer MODULAR P800. Folic acid and vitamin B_12_ levels were estimated by electrochemiluminescence immunoassay on the analyzer MODULAR ANALYTICS E170 (Roche Diagnostics GmbH, Mannheim, Germany) and those for vitamins B_2_ and B_6_ by high performance liquid chromatography (HPLC) with fluorescence detection (Chromsystem Diagnostics GmbH, München, Germany). Estimation of serum homocysteine concentration was carried out by fluorescence polarization immunoassay (AXSYM SYSTEM, Abbott Diagnostics, Mannheim, Germany). Estimation of body weight is made by using the body mass index (BMI) = kg/height(m)^2^.

### 2.3. Statistical Analysis

Differences between patients with and without DR in continuous variables were tested using *t*-test for two independent samples and, when normality assumption was violated, using the Mann-Whitney test. For categorical variables, chi square or Fisher's exact test was applied. Association of homocysteine with folic acid and vitamin B_12_ was investigated using Spearman correlation coefficient, while when HbA_1C_ and diabetes duration groups were considered, one-way ANOVA or Kruskal-Wallis test (with Bonferroni correction when needed) was used. Differences by gender and type of retinopathy were tested by *t*-test for two independent samples or the Mann-Whitney test. The association of homocysteine with possible prognostic variables was also tested in multivariable regression models. This was a two-stage procedure. In the first stage, using generalized additive models (GAMs), the shape of the association with continuous variables was explored. When a threshold was identified, this was estimated using appropriate methodology. In the final regression model, those variables identified in the first step were included in the model using two linear parts, one below and one above the threshold (piecewise linear). Finally, multivariable logistic regression models were used to identify prognostic variables for DR (binary variable, yes/no) in the same way that was described above for homocysteine.

## 3. Results

All our results are presented in median (interquartile range) values. The demographic and laboratory data of the study population are shown in Table 1. The age of patients with DR was statistically higher than that of patients without DR (68.0 (60.0–75.0) versus 61.0 (55.0–67.0), resp., *P* = 0.001), as was also their positive family history of diabetes (86.2% versus 54.7% resp., *P* < 0.001), and their BMI (29.2 (27.9–31.2 versus 27.8 (26.1–30.2), resp., *P* = 0.004). Patients with DR had a significantly longer diabetes duration (15.0 (10.0–22.0) versus 6.0 (3.0–9.0), *P* < 0.001), higher serum glucose (154.0 (126.0–188.0) versus 119.0 (103.0–136.0), *P* < 0.001), HbA_1C_ (7.4 (6.6–8.9) versus 6.7 (6.0–7.6), *P* < 0.001), and serum creatinine level (0.9 (0.8–1.1) versus 0.9 (0.7–1.0), *P* = 0.024), but in both groups creatinine levels were well within the normal range. A significantly higher serum homocysteine level was observed in patients with DR than in those without DR (16.3 (14.7–19.8) versus 11.1 (9.5–13.6), resp., *P* < 0.001), and hyperhomocysteinemia (serum homocysteine >15 *μ*mol/L) was noted in 70.8% of individuals with DR compared to 14.7% without DR (*P* < 0.001). As for the serum vitamins, patients with DR had, though in the normal range, significantly lower folic acid (8.7 (7.2–10.3) versus 11.0 (8.3–17.0), *P* < 0.001) and vitamin B_12_ (288.8 (209.5–416.5) versus 361.5 (255.6–471.2), *P* = 0.014) levels, whereas there was no significant difference for vitamin B_2_ (9.1 (7.0–12.7) versus 10.8 (7.8–13.2), *P* = 0.179) and vitamin B_6_ (11.0 (8.0–14.6) versus 11.6 (9.9–13.2), *P* = 0.481) levels between DR and no DR groups, respectively. Systolic blood pressure was higher in patients with DR (130.0 (120.0–135.0) versus 125.0 (115.0–130.0), *P* = 0.016), but no significant difference in diastolic blood pressure was noted between the two groups (75.0 (70.0–80.0) versus 75.0 (70.0–80.0), *P* = 0.409). However, in all our diabetic patients blood pressure was within the normal range.

In Table 2, the data on the association between serum homocysteine levels and two significant parameters are shown. Serum homocysteine levels of diabetic patients were significantly increased with increasing diabetes duration (≤5 versus ≥16 years, homocysteine: 11.2 (8.7–13.9) versus 16.9 (14.8–18.9), *P* < 0.001, 6–15 versus ≥16 years, homocysteine: 12.2 (10.0–16.2) versus 16.9 (14.8–18.9), *P* = 0.001, overall *P* < 0.001). However, serum homocysteine levels did not change significantly with increments in HbA_1C_ levels (HbA_1C_ < 6.5 homocysteine: 11.2 (9.5–15.8), 6.5–7.4: 12.3 (10.6–18.4), 7.5–8.4: 15.0 (11.1–16.2), ≥8.5: 15.1 (11.3–17.4), *P* = 0.126).

There was a statistically significant difference in serum homocysteine levels between the NPDR group compared to the PDR group (NPDR: 15.5 (11.8–17.4), PDR: 18.7 (16.5–22.0), *P* = 0.001).

A statistically significant negative linear relationship was found between serum homocysteine and folic acid levels (Spearman *r* = −0.261, *P* = 0.002), as well as between serum homocysteine and vitamin B_12_ levels (Spearman *r* = −0.219, *P* = 0.009).

**Figure 1:**
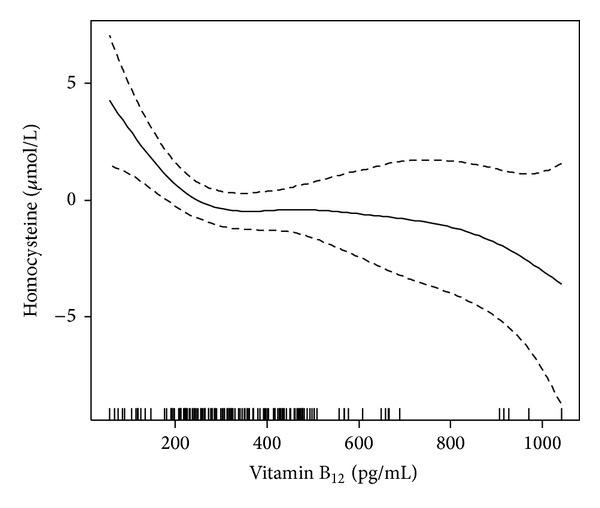
Shape of association between serum homocysteine and B_12_.

Multiple linear regression analysis revealed a significant association between serum homocysteine level and age (Coef.: 0.099, 95% CI: 0.02, 0.18, *P* = 0.018), diabetes duration (Coef.: 0.106, 95% CI: 0.02, 0.20, *P* = 0.020), and an inverse relationship with folic acid (Coef.: −0.220, 95% CI: −0.37, −0.07, *P* = 0.005) and vitamin B_12_ (Coef.: −0.027, 95% CI: −0.04, −0.01, *P* = 0.003). No associations with sex (Coef.: 0.859, 95% CI: −0.56, 2.28, *P* = 0.233), BMI (Coef.: 0.149, 95% CI: −0.06, 0.36, *P* = 0.166), and glucose (Coef.: 0.005, 95% CI: −0.01, 0.02, *P* = 0.523) were observed (Table 3). There is a threshold in the exposure-response association of serum homocysteine with vitamin B_12_, below which changes in serum vitamin B_12_ levels significantly affect serum homocysteine concentration (turning point: 248.4, standard error: 43.8) (Figure 1).

**Figure 2:**
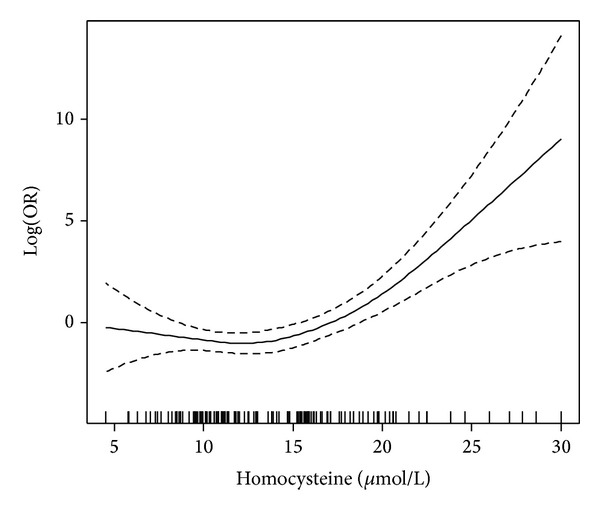
Shape of association between DR risk and serum homocysteine.

Multiple logistic regression analysis also showed that variables that independently affect DR risk were diabetes duration (OR: 1.18, 95% CI: 1.08, 1.28, *P* < 0.001), HbA_1C_ (OR: 2.30, 95% CI: 1.49, 3.54, *P* < 0.001), and homocysteine concentrations (OR: 1.66, 95% CI: 1.24, 2.23, *P* = 0.001). Folic acid conferred a protective effect on the DR risk (OR: 0.73, 95% CI: 0.62, 0.86, *P* < 0.001) (Table 4). There is a threshold in the association of homocysteine with DR (turning point: 13.7, standard error: 1.4). For every increase of serum homocysteine by 1 *μ*mol/L above the threshold, there is an increased risk of about 66% for the development of DR (Figure 2).

## 4. Discussion

The results of our study revealed significantly higher concentration of serum homocysteine as well as a higher prevalence of hyperhomocysteinemia (serum homocysteine > 15 *μ*mol/L) in patients with T2DR compared to those without DR and hyperhomocysteinemia as an independent risk factor for DR. Our results also revealed a threshold in the association of serum homocysteine level with DR risk of around 14.0 *μ*mol/L, above which a 1 *μ*mol/L increase in serum homocysteine concentration was an independent predictor of increased DR risk of 66%. A higher serum homocysteine level in patients with PDR than in those with NPDR was also found; a result that may suggest that homocysteine is related not only to the development but also to the progression of DR. There are a number of studies that have evaluated the association between hyperhomocysteinemia and type 2 DR but have so far yielded inconsistent results [6–9]. The differences in the findings among studies are most likely related to differences in type of diabetes or to inadequately controlling for confounding factors, such as nephropathy and hypertension, but also lifestyle (e.g., vitamin intake) and genetic factors. In addition, the cutoff level for hyperhomocysteinemia differed substantially among studies (from 11.0 to 16.0 *μ*mol/L).

The exact pathogenesis of DR is multifactorial and remains largely unclear but may involve (1) endothelial dysfunction and (2) low-grade chronic inflammation of the retinal capillaries. Hyperhomocysteinemia promotes these two pathophysiologic mechanisms [10]. It has long been recognized that oxidative stress is associated with the progression of diabetes and its complications. The adverse effects of hyperhomocysteinemia on the endothelium may be triggered by increased oxidative stress in the diabetic vasculature. Hyperhomocysteinemia increases NADPH oxidase activity [11], promotes uncoupling of endothelial nitric oxide synthase [12], and inhibits the function of intracellular antioxidant enzymes, such as glutathione peroxidase and superoxide dismutase [13]. Moreover, autooxidation of excess homocysteine may directly lead to additional ROS production [14]. Accumulating ROS reacts with nitric oxide (NO) to form peroxynitrite radicals, leading to decreased NO bioavailability and activity and subsequent endothelial dysfunction.

ROS activate inflammatory mediators and cytokines (interleukins, tumor necrosis factor-*α*). In addition, upregulation of ROS leads to activation of proinflammatory mediators, such as the nuclear factor-*κΒ*, in retinal capillary cells, which further increasing the expression of cytokines [15]. These inflammatory events result in increased endothelial cell expression of adhesion molecules, which contribute to leucocyte accumulation and attachment to the retinal capillaries (leucostasis) [16]. Leucostasis, which is believed to be an early event in DR, may lead to the blood-retinal barrier breakdown and eventually to chronic leucocyte mediated cell damage and death [17].

With regard to the risk factors for DR, regression analysis of our data confirmed that poor glycemic control and longer diabetes duration are independent risk factors for the development and progression of DR [18, 19]. In our study, the group of patients without DR succeeded in achieving the target mean HbA_1C_ level of  7.0% or less [4] (HbA_1C_6.9 ± 1.1%) but this was not the case with the group of patients with DR (HbA_1C_  7.8 ± 1.5%). However, it is true that perfect glycemic control is very difficult to attain for most diabetic patients and glucose control has the tendency to deteriorate with time. A significant finding in our study was the association of increasing serum homocysteine concentration with longer diabetes duration. Thus, we may infer that the known harmful effect of DM duration on the development of retinopathy and its progression is not only related to the long-standing hyperglycemia but also to the increased homocysteine concentrations with time and its subsequent adverse effects on retinal capillaries. On the contrary, we did not find an association between homocysteine concentrations and increasing HbA_1C_ levels. This may be due either to an actual lack of such an association between the two factors or to the inconsistency of hyperglycemia during the progression of DM. Besides, evidence from both healthy and diabetic twins indicates that HbA_1C_ levels are determined for a significant part by genetic factors. This provides evidence that HbA_1C_ is in part determined by factors other than glycemic control and may account for the variation in HbA_1C_ levels among people [20, 21].

Methionine and homocysteine metabolism depends upon adequate stores of folic acid and vitamins B_2_, B_6_, B_12_, which act as cofactors or substrates in the metabolism and are important nutritional determinants of serum homocysteine. A number of studies have shown an inverse association between blood folic acid and homocysteine concentration [22, 23]. Such an inverse relationship between folic acid and homocysteine levels was also observed in our study. In regard with vitamin B_12_, the reason for the limited number of studies with this vitamin is that its deficiency is rather unusual, especially in Western countries, due to the fact that the vitamin is present in food of animal origin. A recent study from India, where the prevalence of micronutrient deficiency is reported to be high, mentions a high prevalence of vitamin B_12_ deficiency in the diabetic population (54–67%). Mean vitamin B_12_ level in the DR group was below the normal range and higher homocysteine levels were significantly associated with lower vitamin B_12_ levels [24]. We also detected such an inverse association between vitamin B_12_ and homocysteine levels. Our study illustrates lower levels of folic acid and vitamin B_12_ in patients with DR. It is noteworthy that low, but not necessarily deficient, levels of these two vitamins contributed to the development of hyperhomocysteinemia in our study. Regression analysis also showed that folic acid confers a protective effect on the development of DR. Another significant observation in our study was the finding of a threshold of about 250 pg/mL of serum vitamin B_12_, below which there is a sharp rise in serum homocysteine with decreasing vitamin B_12_ levels.

Vitamin B_6_ deficiency is extremely unusual, because this vitamin is present in a wide range of food of animal and plant origin. In our study, we found no cases of biochemical vitamin B_6_ deficiency. One study has reported an association between low vitamin B_6_ and vascular disease that was unrelated to hyperhomocysteinemia, but it was explained by a relationship between low vitamin B_6_ status and chronic inflammation, which is known to promote DR [25]. The results of our study show that vitamin B_6_ levels were well within the normal range in patients with diabetes irrespective of the presence of retinopathy. Vitamin B_2_ acts in the form of flavin adenine dinucleotide (FAD), which is a substrate for the enzyme methylene tetrahydrofolate reductase (MTHFR). This enzyme is the most important genetic determinant of blood homocysteine in the general population. In our study material, no cases of biochemical vitamin B_2_ deficiency were observed, neither do we know any such studies, and the levels of this vitamin were also well within the normal range in all of our diabetic patients. Increased dietary folic acid and vitamin B_2_ requirements have been observed mainly in persons with the genetic variant of the enzyme MTHFR, C677T [26]. Studies confirm that vitamin B_2_ is an independent determinant of homocysteine in TT homozygotes of the C677T polymorphism [27].

In conclusion, among the many factors, some of them are yet unidentified, that affect the development and progression of DR, hyperhomocysteinemia seems to be associated with DR, at least as a biomarker. Longer diabetes duration and lower folic acid and vitamin B_12_ status appear to be important determinants for hyperhomocysteinemia in DR patients. Monitoring serum homocysteine concentration, as well as folate and vitamin B_12_ status in T2DM patients, could be used as an indicator for assessing microvascular risk in DM. Treatment of existing hyperhomocysteinemia with folic acid and vitamin B_12_ may be useful in reducing the risk of microvascular complications in T2DM.

## Figures and Tables

**Table 1:** Demographic and laboratory characteristics of patients with type 2 diabetes mellitus.

Characteristics	DR− (*N* = 75)	DR+ (*N* = 65)	*P* value
Age (years)	61.0 (55.0–67.0)	68.0 (60.0–75.0)	0.001
Positive family history (%)	54.7 (*N* = 41)	86.2 (*N* = 56)	<0.001
BMI (kg/m^²^)	27.8 (26.1–30.2)	29.2 (27.9–31.2)	0.004
Diabetes duration (years)	6.0 (3.0–9.0)	15.0 (10.0–22.0)	<0.001
Fasting serum glucose (mg/dL)	119.0 (103.0–136.0)	154.0 (126.0–188.0)	<0.001
HbA_1C_ (%)	6.7 (6.0–7.6)	7.4 (6.6–8.9)	<0.001
Serum creatinine (mg/dL)	0.9 (0.7–1.0)	0.9 (0.8–1.1)	0.024
Serum homocysteine (*μ*mol/L)	11.1 (9.5–13.6)	16.3 (14.7–19.8)	<0.001
Hyperhomocysteinemia (%)	14.7 (*N* = 11)	70.8 (*N* = 46)	<0.001
Serum folic acid (ng/mL)	11.0 (8.3–17.0)	8.7 (7.2–10.3)	<0.001
Serum vitamin B_2_ (*μ*g/L)	10.8 (7.8–13.2)	9.1 (7.0–12.7)	0.179
Serum vitamin B_6_ (*μ*g/L)	11.6 (9.9–13.2)	11.0 (8.0–14.6)	0.481
Serum vitamin B_12_ (pg/mL)	361.5 (255.6–471.2)	288.8 (209.5–416.5)	0.014
Systolic blood pressure (mmHg)	125.0 (115.0–130.0)	130.0 (120.0–135.0)	0.016
Diastolic blood Pressure (mmHg)	75.0 (70.0–80.0)	75.0 (70.0–80.0)	0.409

Values are presented as median (interquartile range) or % (number).

**Table 2:** Association of serum homocysteine levels with diabetes duration and glycosylated hemoglobin.

Parameters		Homocysteine (*μ*mol/L)	
Diabetes duration (years)	≤5 (*N* = 42)	11.2 (8.7–13.9)	*P* value*
	6–15 (*N* = 60)	12.2 (10.0–16.2)	
	≥16 (*N* = 38)	16.9 (14.8–18.9)	
	Overall (*N* = 140)	12.9 (10.3–16.9)	
HbA_1C_ (%)	<6.5 (*N* = 43)	11.2 (9.5–15.8)	*P* value**
	6.5–7.4 (*N* = 43)	12.3 (10.6–18.4)	
	7.5–8.4 (*N* = 26)	15.0 (11.1–16.2)	
	≥8.5 (*N* = 28)	15.1 (11.3–17.4)	
	Overall (*N* = 140)	12.9 (10.3–16.9)	

*≤5 years versus ≥16  *P* < 0.001, 6–15 years versus ≥16 *P* = 0.001, overall  *P* < 0.001.

**P = 0.126.

Values are presented as median (interquartile range).

**Table 3:** Multiple linear regression analysis of the association of serum homocysteine with independent variables.

	Coef.	95% CI	*P* value
Age (years)	0.099	0.02, 0.18	0.018
Sex (male)	0.859	−0.56, 2.28	0.233
BMI (kg/m²)	0.149	−0.06, 0.36	0.166
Fasting serum glucose (mg/dL)	0.005	−0.01, 0.02	0.523
Diabetes duration (years)	0.106	0.02, 0.20	0.020
Folic acid (ng/mL)	−0.220	−0.37, −0.07	0.005
Vitamin B_12_^†^ (pg/mL)	−0.027	−0.04, −0.01	0.003
Vitamin B_12_^‡^ (pg/mL)	−0.002	−0.01, 0.00	0.409

^†^below threshold.

^‡^above threshold.

**Table 4:** Multiple logistic regression analysis of the association of diabetic retinopathy with prognostic variables.

	OR	95% CI	*P* value
Age (years)	1.03	0.97–1.09	0.332
Diabetes duration (years)	1.18	1.08–1.28	<0.001
HbA_1C_ (%)	2.30	1.49–3.54	<0.001
Homocysteine^†^ (*μ*mol/L)	1.66	1.24–2.23	0.001
Homocysteine^‡^ (*μ*mol/L)	0.83	0.61–1.12	0.219
Folic acid (ng/mL)	0.73	0.62–0.86	<0.001

^†^above threshold.

^‡^below threshold.
